# Square Split-Ring Resonator as a Sensor for Detection of Nanoparticles in PVDF-Based Nanocomposites at Ultra-High Frequencies: MXenes and MoS_2_ Concentrations

**DOI:** 10.3390/s26031028

**Published:** 2026-02-04

**Authors:** Jorge Simon, Jacobo Jimenez-Rodriguez, Emmanuel Hernandez-Gonzalez, Jose L. Alvarez-Flores, Walter A. Mata-Lopez, John A. Franco-Villafañe, J. R. Gomez-Rodriguez, Marco Cardenas-Juarez, Oscar F. Olea-Mejia, Ana L. Martinez-Hernandez, Carlos Velasco Santos

**Affiliations:** 1 Facultad de Ciencias, Universidad Autónoma de San Luis Potosí, San Luis Potosí 78295, Mexico; 2 Facultad de Química, Universidad Autónoma del Estado de México, Toluca 50130, Mexico; 3 Unidad Académica de Ingeniería Eléctrica, Universidad Autónoma de Zacatecas, Zacatecas 98000, Mexico; 4 Facultad de Ingeniería Mecánica y Eléctrica, Universidad de Colima, Colima 28040, Mexico; 5 Instituto de Física, Universidad Autónoma de San Luis Potosí, San Luis Potosí 78295, Mexico; 6 Centro Conjunto de Investigación en Química Sustentable, Universidad Autónoma del Estado de México, Toluca 50200, Mexico; 7 División de Posgrado e Investigación, Campus Querétaro, Tecnológico Nacional de México, Querétaro 76017, Mexico

**Keywords:** square split-ring resonator, sensor, nanoparticles, PVDF-based nanocomposites, UHF

## Abstract

The performance of a printed square split-ring resonator as a sensor for quantifying nanoparticle concentrations in PVDF-based nanocomposites was evaluated at UHF frequencies. The sensing mechanism was based on the frequency response of parameter S_21_, observing the shift in the resonant frequency and a variation in S_21_ level, while samples were placed on the ring split and compared to the sensor without a sample. Experiments with samples of PVDF-based nanocomposites combined with different concentrations of both MoS_2_ and MXenes, ranging from 0.01% to 0.2%, were conducted. In general, considering both types of samples studied, it was observed that, as the concentration increases, S_21_ (dB) increases from −6.35 to −6 dB. At the same time, the resonance frequency in the S_21_ plot went from 500.4 to 498.25 MHz. Although the concentrations and their variations were relatively low, shifts in the resonance frequency of S_21_ were evident, demonstrating the ability of the sensor to detect low concentrations and variations of MoS_2_ and MXenes, being the detection of samples with higher concentrations feasible as future work, and concluding that the sensor had a relatively acceptable performance. In this study, MXenes were the concentrations that produced more noticeable shifts in the resonance frequency of S_21_. Likewise, characterizations based on SEM and TEM were performed to corroborate the ones at UHF frequencies.

## 1. Introduction

Nanoparticles (NPs) have reached a significant impact across multiple scientific disciplines due to their unique physicochemical properties and wide-ranging applications [1]. Incorporating NPs into polymer matrices has led to the development of nanocomposites with enhanced mechanical, electrical, and thermal properties [2]. Among these, polyvinylidene fluoride (PVDF)-based nanocomposites have appeared as particularly promising materials for diverse applications, including energy storage, sensors, flexible electronics, and biomedical devices [3].

The dielectric properties of these materials play a critical role, as they directly influence the S-parameters of devices such as sensors that incorporate them [4,5,6,7]. For instance, combinations of PVDF with MXenes or silver nanowire networks have been successfully applied in high-performance electromagnetic interference (EMI) shielding and efficient heat dissipation, increasing the need for precise determination of nanoparticle concentration within PVDF-based nanocomposites [8]. However, accurate and efficient quantification of NP concentration in such composites remains a major challenge.

Conventional characterization techniques, such as atomic force microscopy (AFM) and transmission electron microscopy (TEM), scanning electron microscopy (SEM) or other optical methods, while capable of providing high-resolution imaging, are inherently time-consuming, expensive, destructive or semi-destructive, and unsuitable for real-time or in situ monitoring during fabrication or quality control [9,10].

In contrast, microwave-based sensing using split-ring resonators (SRRs) offers a fundamentally different and highly advantageous approach. Operating in the ultra-high frequency (UHF) range, SRR-based sensors provide non-destructive and contactless measurement, allowing repeated characterization of the same sample without any physical or chemical alteration. They deliver rapid results, typically within seconds to minutes, through straightforward S-parameter measurements, in contrast to the hours or days required by microscopy-based methods. Moreover, SRR can be fabricated using low-cost, planar microwave circuitry, offering the potential for compact, portable, or even handheld systems suitable for in-line process monitoring [11,12,13,14]. Their most distinctive feature lies in the strong confinement of the evanescent electric field within the narrow split-gap region, which results in exceptional sensitivity to subtle changes in the local dielectric environment induced by variations in nanoparticle concentration, type, size, shape, and spatial distribution within the PVDF matrix. Compared to other microwave sensing techniques, such as simple transmission-reflection lines, dielectric cavity resonators, or non-resonant waveguide methods, SRRs are particularly advantageous due to their sharp resonance features, high quality factor (Q), compact size, and pronounced response to localized dielectric perturbations in the gap area [15,16,17,18,19,20,21,22].

The innovative aspect of the present work lies in the direct placement of solid PVDF-based nanocomposite samples onto the split of a common SRR geometry, enabling sensitive near-field probing of the interaction between the resonator and the embedded nanoparticles, an approach that remains underexplored for the quantitative characterization of polymer nanocomposites at UHF frequencies.

This study introduces a novel application of an SRR-based microwave sensor for the detection and quantification of nanoparticle concentrations in PVDF-based nanocomposites. It investigates the relationship between the transmission parameter S_21_ (including resonance frequency shift and depth) and the NP concentration in samples positioned on the SRR split. By systematically characterizing the sensor response across a narrow range of concentrations, this work aims to establish a reliable correlation for accurate quantitative determination of nanoparticle loading. Furthermore, the influence of NP type, size, shape, and distribution on sensor performance is examined with the support of SEM and TEM to elucidate the underlying sensing mechanisms. The proposed SRR-based sensors offer a promising pathway toward rapid, non-destructive, and potentially in situ monitoring of nanoparticle concentrations in PVDF-based nanocomposites, which could significantly enhance quality control, process optimization, and performance reliability during fabrication and practical application.

## 2. Materials and Methods

For the characterization of PVDF-based nanocomposite samples, an experimental setup implying a printed square SRR connected to a two-port vector network analyzer (VNA), later described in Section 2.8, was considered for measuring two-port S-parameters (scattering parameters). The measurement was performed before and after disturbance caused by the presence of the PVDF-based nanocomposite samples, which were prepared to match the geometry of the SRR split, specifically, having them on the ring split and taking the form of a disk to cover the SRR rectangular split. Likewise, a numerical analysis using the academic version of Ansys Electronics Desktop^®^ (AEDT) [23] that implements the finite element method was performed to compare simulation against measurement results. Finally, SEM and TEM were performed to structurally analyze and correlate sample surface morphology changes with changes in S-parameters of the sample-containing SRRs [24].

Next, the steps involved in the methodology of this work are described:

### 2.1. Selection of Materials

The materials involved in the manufacture of the samples and their corresponding characteristics are listed as follows:

- Ti_3_AlC_2_, purity: 99%, APS: 40–60 μm (MAX Phase, Titanium Aluminum Carbide Powder).
- PVDF with a density of 1.74 g/mL at 25 °C and an average molecular weight of ~534,000 (as determined by GPC), powder form.
- Hydrofluoric acid (HFA), 48%.
- Molybdenum (IV) sulfide (MoS_2_) with a particle size of ~6 μm, powder.
- Isopropyl alcohol (≥99.5%).
- Methanol with a purity of ≥99.8%.
- Dimethyl sulfoxide with a purity of ≥99.9%.
- Plasticizer Scona TPPE 1102 PALL, BYK Kometra GmbH, Schkopau, Germany, a maleic anhydride-modified, carboxylated, linear low-density polyethylene.

### 2.2. Preparation of MXene Nanosheets

The MXene nanosheets were obtained through a selective etching [25], intercalation, and delamination method, as reported in some previous studies [26,27]. To conduct the synthesis of MXenes, a selective etching process was performed following this procedure: initially, a 15% hydrofluoric acid (HF) solution was prepared, and gradually, 1 g of Ti_3_ AlC_2_ powder (MAX phase) was added to it. The mixture was continuously stirred for 24 h [28] at a temperature of 35 °C [29] using a magnetic stirrer. Subsequently, the resulting solution was washed with deionized water. In total, 50 mL of deionized water was added to the mixture, and 5-min centrifugation cycles were performed, repeating this process several times until the pH approached 6. The supernatant was then discarded, and the moist powders were retained for the subsequent synthesis step. The intercalation of MXenes was carried out by dispersing the multilayer MXene powders in 40 mL of dimethyl sulfoxide (DMSO) [27]. The mixture was stirred for 18 h to facilitate the intercalation of DMSO between the layers of the MXenes. Once the intercalation was completed, excess DMSO was removed by washing with deionized water, involving centrifugation cycles at 3500 rpm for 15 min. To achieve the delamination of the MXenes, 30 mL of deionized water was added to the moist powders obtained after intercalation. The mixture was sonicated for 1 h using an ultrasound probe in a cold bath. Finally, the MXene material (Ti_3_C_2_T_x_) was dried in an oven at 65 °C for subsequent use.

### 2.3. Preparation of MoS_2_ Nanosheets

MoS_2_ nanosheets were obtained through the liquid-phase exfoliation (LPE) method [27]. Specifically, 100 mg of MoS_2_ powder was dissolved in a 100 mL glass beaker with 50 mL of isopropyl alcohol (IPA). The MoS_2_ was exfoliated using ultrasound with an ultrasound probe for 8 h in cold baths and at 60% amplitude. The excess solvent was evaporated in an oven at 65 °C, and the obtained powder was stored for future use.

### 2.4. Preparation of Extruded Nanocomposites

The nanocomposites were fabricated using a process that involved dissolution, mixing, drying, and extrusion. To begin, 50 g of PVDF powder and 1 g of plasticizer Scona TPPE 1102 Pall powder were slowly dispersed in 150 mL of water while continuously stirring using a magnetic stirrer. The nanoparticles were weighed separately. The MXene particles were dispersed in 40 mL of water and subjected to a 30-min sonication process in a cold bath using an ultrasound probe (Cole-Parmer, Vernon Hills, IL, USA) with an amplitude of 50%. Similarly, MoS_2_ nanoparticles were dispersed in 40 mL of IPA and subjected to the same sonication conditions as the MXenes particles. The nanoparticle dispersions were gradually incorporated into the PVDF mixture, which was continuously stirred for 30 min at room temperature to ensure a homogeneous mixture. Subsequently, the solvent was removed from the homogeneous mixture through a drying process in an oven at 65 °C. The resulting dry powders were extruded using a Filabot^®^ extruder at a temperature of 260 °C, allowing the production of filaments with a diameter of 1.75 mm. These filaments were preserved for future use.

### 2.5. Fabrication of Disk-Shaped Samples for Characterization of S-Parameters

**Figure 1:**
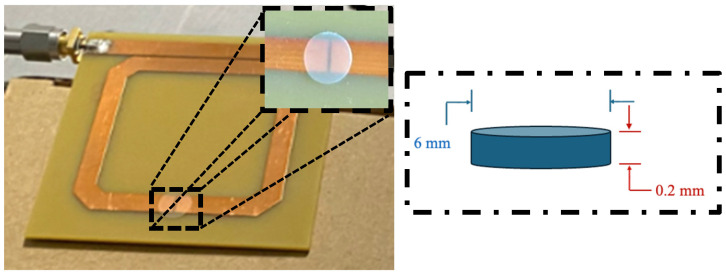
Disk-shaped sample on the split of the SRR.

To conduct the characterization of S-parameters in the presence of the PVDF-based composites, it was necessary to fabricate the specimens (samples) designed to match the experimental setup based on an SRR. To create the samples to be located on the split of the SRR, two five cm-long segments of filament-like composites were sandwiched between two glass sample plates measuring 25 mm × 75 mm. The glass plates, holding the filament segments, were secured using six binder clips and then heated in an oven at 230 °C until the compound melted, forming a thin film. After cooling, the films were detached from the glass plates and cut using a hole punch to obtain disk-shaped samples with a diameter of 6 mm and an approximate thickness of 0.20 mm. Figure 1 shows the disk-shaped sample on the ring split.

### 2.6. SEM Analysis

To prepare polymer composites for analysis with an SEM, they were first cut into small sections and cleaned to remove contaminants. Then, they were mounted on copper stubs using double-sided carbon tape as an adhesive. Subsequently, the samples were coated with a thin layer of gold via sputtering, with the sputter coater parameters adjusted to achieve a uniform coating. Finally, the samples were examined under the microscope to study surface morphology and analyze the differences between the composites at various nanomaterial concentrations.

### 2.7. TEM Analysis

TEM was employed to investigate the morphology and structural features of the exfoliated MXene and MoS_2_ nanosheets. Diluted dispersions of the nanomaterials were drop-cast onto carbon-coated copper grids and dried under ambient conditions. TEM observations were conducted to qualitatively assess the lamellar morphology, sheet-like structure, and local stacking behavior of the nanosheets.

### 2.8. The SRR and the Measurement of S-Parameters

**Figure 2:**
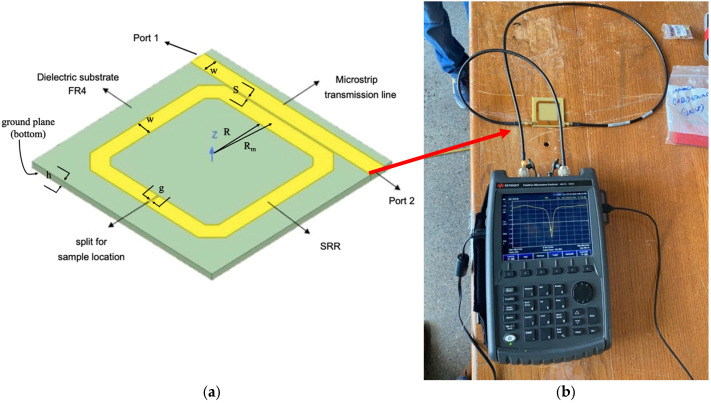
(**a**) Schematic of the SRR and (**b**) a top view of the setup for the measurement of parameter S_21_ using an N9917A.

An SRR was implemented through printed circuit board (PCB) technology on an FR4 substrate using a milling machine. The printed SRR is **square-shaped** with flattened corners and coupled to a straight microstrip transmission line (MTL) including 50 Ω female SubMiniature version A (SMA) ports (connectors) at each of its two ends. The FR4-based PCB was double-sided, with the bottom assigned to be the circuit ground plane. The SRR includes a split where samples are located to characterize the corresponding materials under test (MUTs). Figure 2 shows (a) a schematic of the SRR and (b) a top view of the experimental setup used to measure parameter S_21_ for the SRR with a sample (disk-shaped) on the ring split, while Table 1 shows SRR dimensions. The measurement of the S-parameter (S_21_) was conducted at the two 50 Ω female SMA ports of the PCB connecting two 1 m-long 50 Ω male-to-male SMA coaxial pigtail cables, one coming from the N9917A VNA (Keysigth Technologies, Santa Rosa, CA, USA) [30] and the other returning to the same instrument. This connection allows us to get S_21_ as a function of frequency, which is the transmission coefficient between ports. Measurement of S_21_, including each of the samples under test (SUTs), was performed at UHF frequencies from 400 to 600 MHz, considering 601 values to find the frequency at which the measured resonance (f_MR_) occurs and use it as the frequency to characterize the SRR as a sensor. The measurements were performed after a two-port SOLT (short-open-load-through) calibration using an 85521A SMA calibration kit (Keysigth Technologies, Santa Rosa, CA, USA) [31]. Finally, it is worth mentioning that, from the two 50 Ω female type-N ports of the N9917A to the two 50 Ω SMA cables, two female type-N to male SMA coaxial adapters were included.

### 2.9. SRR and Lumped-Element Model

The square SRR implemented in this work, whose dimensions are shown in Table 1, was based on the one used as an aptamer-based biosensor and reported by [32]. The structure implemented in this work has a parameter S_21_ showing a measured resonance at f_MR_ = 500.4 MHz and a simulated resonance at f_SR_ = 499.2 MHz, as both will be shown later in Figure 12. Note that f_MR_ at 500.4 MHz will be used as the frequency to characterize the SRR as a sensor when having samples of materials under test on the split, causing a frequency shift.

Square SRRs, like all microstrip circuits fabricated as PCBs, have a lumped-element equivalent circuit. To get that equivalence, the mathematical expressions for the LC (inductance L_S_-capacitance C_S_) model and the resonance frequency f_RL_ were taken from [33] through Equations (1)–(9) as follows:

(1) $$f_{RL}=\frac{1}{2\pi \sqrt{L_{S}C_{S}}}.$$

**Figure 3:**
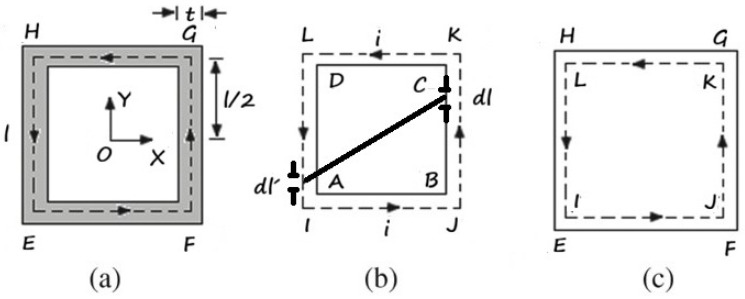
(**a**) 2D geometry, (**b**) the inner edge ABCD, and the (**c**) outer edge EFGH of the SRR.

**Figure 4:**
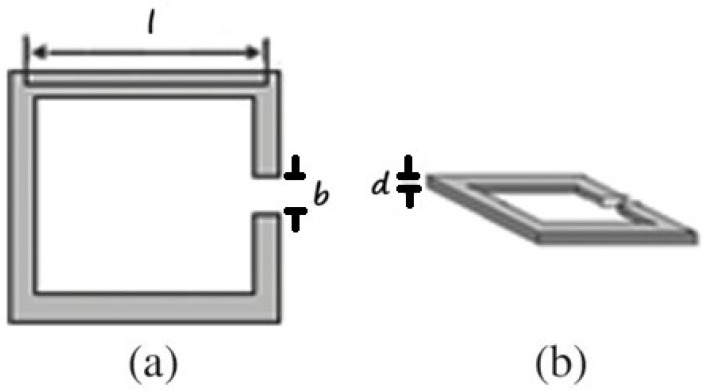
Two different views of the SRR and its dimensions: (**a**) top view and (**b**) oblique view.

The inductance L_S_ is computed in terms of the mean value of the magnetic flux “Φ“ through the loop of the SRR due to a constant current “i” supposed to flow along the center line as shown in Figure 3, where (a) is the 2D SRR geometry, (b) is the inner edge (ABCD), and (c) the outer edge (EFGH), while Figure 4 shows two different views of the SRR, as well as the dimensions involved in the design.

ABCD and EFGH represent the inner and the outer edges of the loop, respectively, so that

(2) $$L_{s}=\frac{\Phi }{i}=\frac{1}{2i}\left(\Phi_{ABCD}+\Phi_{EFGH}\right)$$

To calculate “Φ”, standard formulas implying vector calculus and electrodynamics were applied for the loops either ABCD or EFGH as follows:

(3) $$\Phi =\iint \vec{B}\times \vec{dS}=\iint (\vec{\nabla }\times \vec{A})\times \vec{dS}=\oint \vec{A}\times \vec{dl},$$

where B stands for magnetic flux, and A represents the corresponding vector potential. The approximate value for inductance L_s_ is the following:

(4) $$L_{s}=\frac{2\mu_{0}}{\pi }\left[\ln\left(l\right)-\ln\left(t\right)+\{\sqrt{2}+2\ln\left(2\right)-\ln\left(\sqrt{2}+1\right)-2\}\right]l.$$

The total capacitance of the SRR C_S_ = C_gap_ + C_surf_, where C_gap_ is the gap capacitance and is related to the capacitance of a parallel-plate capacitor formed in the gap or split region of the SRR, while the surface capacitance C_surf_ considers the fringe-field effect. C_gap_ and C_surf_ are defined considering Equations (4)–(9):

(5) $$C_{gap}=\frac{\varepsilon_{o}\cdot \varepsilon_{r,air}\cdot d\cdot t}{b}+\varepsilon_{o}\cdot \varepsilon_{r,air}\cdot \left(d+b+t\right)$$

(6) $$C_{surf}=2\left(d+t\right)\cdot \varepsilon_{r,sub}\cdot C_{surf}^{pul}=2\left(d+t\right)\cdot \varepsilon_{r,sub}\cdot \left(I_{x}+I_{y}-I_{x}^{\prime }\right)$$

(7) $$I_{x}=\int_{b/2}^{l/2}\frac{\varepsilon_{0}}{2l-x}\left[\frac{l}{2x}+\sqrt{{\left(\frac{l}{2x}\right)}^{2}+1}\right]dx$$

(8) $$I_{y}=\int_{0}^{l}\frac{\varepsilon_{0}}{\left(3/2\right)l-y}\left[\frac{l-2y}{l}+\sqrt{{\left(\frac{l-2y}{l}\right)}^{2}+1}\right]dy$$

(9) $$I_{x}^{\prime }=\int_{l/2}^{\delta /2}\frac{\varepsilon_{0}}{x}\left[\frac{-l}{2x}+\sqrt{{\left(\frac{-l}{2x}\right)}^{2}+1}\right]dx.$$

**Figure 5:**
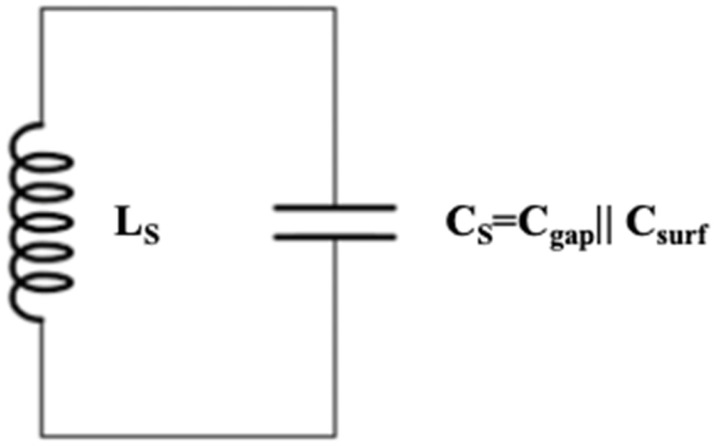
Lumped-element model of any circular SRR (without MTL).

Once the inductance L_S_ and the total capacitance C_S_ are found, the equivalent LC circuit for the SRR can be represented. Figure 5 shows the lumped-element model of any SRR without MTL.

Regarding the forenamed SRR geometry, it is important to consider that a square SRR was chosen (based on [32]) because they are relatively easier to manufacture, either using a chemical technique (etching) or a mechanical technique (milling). In addition, the square ring has a longer section for edge-coupling with MTL compared with circular rings. Applying design equations to the squared geometry in Figure 2a, the resonance frequency for the lumped equivalent would be f_RL_ = 505.96 MHz. These results show that f_MR_ = 500.4 MHz is very similar to f_RL_, and it is the lowest resonant band for the microstrip circuit to characterize the SRR as a sensor at UHF frequencies, a band of frequencies very common in LoRa standards and sensors for IoT (Internet of Things) [34].

**Figure 6:**
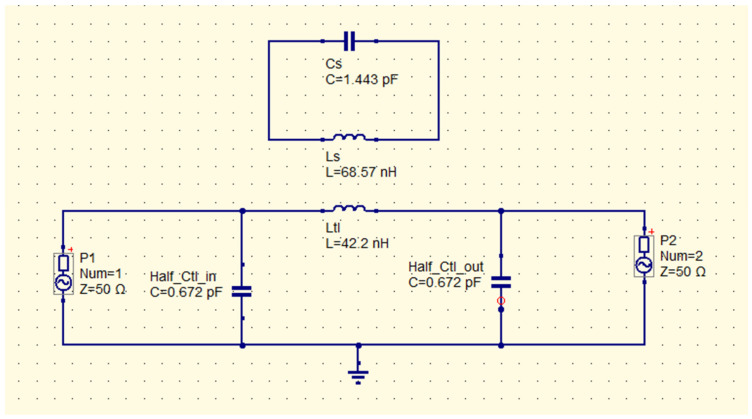
LEC of the SRR coupled to the MTL with resonance frequency f_RL_ = 505.96 MHz.

**Figure 7:**
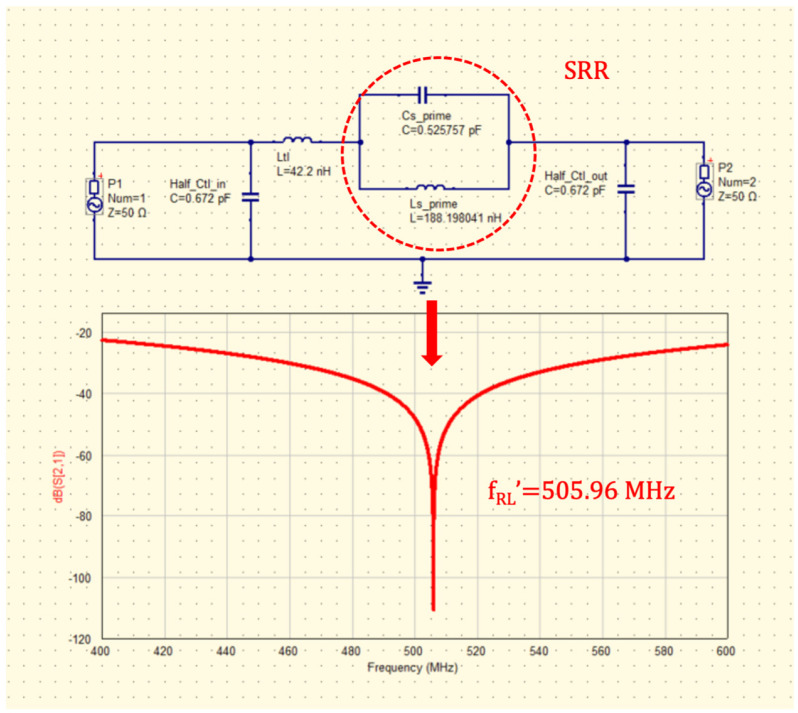
LESC of the SRR coupled to the MTL with resonance frequency f_RL_′ = 505.96 MHz.

For the printed SRR without MTL, the lumped-element equivalent circuit can be modeled as an LC-tank circuit (Figure 5) formed by parameters such as an inductance L_S_ and a capacitance C_S_, which depend on materials and the geometry used to design the SRR. The material used for the SRR substrate was FR4, which has a relative permittivity ε_r_ = 4.4, while dimensions related to the geometry are those shown in Table 1. The PCB is double-sided as it incorporates a ground plane on the bottom, and the copper thickness is t = 0.035 mm. To model the SRR using lumped elements, let us find the inductance L_tl_ and the capacitance C_tl_ corresponding to the straight MTL coupled to it, to have the complete lumped-element circuit (LEC) for what is shown in Figure 2a. The MTL dimensions are 60 mm × 1.5 mm, which give L_tl_ = 42.2 nH according to [35] and C_tl_ = 1.344 pF based on [36]. According to [37], the LEC for the PCB corresponding to the schematic in Figure 2a is shown in Figure 6. LEC is formed by L_S_ = 68.57 nH and C_S_ = 1.443 pF corresponding to the SRR, as well as L_tl_ = 42.2 nH and C_tl_ = 1.344 pF corresponding to the MTL. In Figure 6 and Figure 7, C_tl_ is divided by 2, and each half of it is included at the input (in) and output (out) ports.

The circuit in Figure 6 includes mutual inductance L_M_ between L_S_ and L_tl_. Instead of that, another more practical lumped-element simplified circuit (LESC) shown in Figure 7 is found [37]. This LESC implies some more calculations to get even C_e_ (ε_r_ = 1) and odd C_o_ (ε_r_ = 1) capacitances, both by means of even Z_e_ and odd Z_o_ impedances caused by edge-coupled microstrips (a portion of the MTL coupled to the upper horizontal part of the SRR). Based on [38,39,40], Z_e_ = 38.1 Ω and Z_o_ = 30 Ω, impedances that help to find C_e_ and C_o_, which in turn helped to find the parameter L_M_. The calculations are shown below:

(10) $$C_{e}=\frac{\sqrt{\varepsilon_{r}}}{cZ_{e}}=\frac{1}{c(38.1\;\Omega )}=87.49\;\text{pF}$$

(11) $$C_{o}=\frac{\sqrt{\varepsilon_{r}}}{cZ_{o}}=\frac{1}{c(30\;\Omega )}=111.11\;\text{pF}$$

(12) $$L_{M}=\frac{\mu_{0}\varepsilon_{0}}{2}\left(\frac{1}{C_{e}}+\frac{1}{C_{o}}\right)=113.6\;nH.$$

Once C_e_, C_o_, and L_M_ are known, alternative parameters C_s_′ = 12.9972 pF and L_s_′ = 7.7304 nH as part of the LESC were calculated. L_s_′ and C_s_′ also cause a resonance f_RL_′ = 505.96 MHz as LEC in Figure 6 does. P_1_ and P_2_ in Figure 6 and Figure 7 represent the 50 Ω input and output ports of the networks LEC and LESC. Below, Table 2 shows the resonances for LEC and LESC shown in Figure 6 and Figure 7, while Equations (11) and (12) were used to calculate C_s_′ and L_s_′. Simulations of LEC and LESC were carried out using QUCS (Quite Universal Circuit Simulator) [41].

(13) $$C_{s}^{\prime }=\frac{L_{S}}{{L_{M}}^{2}{\omega_{0}}^{2}}=\frac{68.57\;nH}{{\left(113.6\;nH\right)}^{2}{\left(2\pi (505.96\;MHz)\right)}^{2}}=0.525\;pF$$

(14) $$L_{s}^{\prime }=C_{S}{L_{M}}^{2}{\omega_{0}}^{2}=\left(1.443\;pF\right){\left(113.6\;nH\right)}^{2}{\left(2\pi (505.96\;MHz)\right)}^{2}=188.198\;nH$$

### 2.10. Simulation of S-Parameters for the SRR

Simulation of S-parameters for the SRR was performed using the academic version of AEDT [23], which in turn is a multipurpose, full-wave 3D electromagnetic simulation software based on the finite element method. Regarding the simulation steps, first, the MTL-coupled SRR was drawn using the computer-aided design (CAD) tool included in AEDT. Once the SRR was drawn, including the transmission line and the substrate, some boundary conditions and materials were defined. Boundary conditions were perfect electric conductors (PEC) for the printed SRR, the transmission line, and the ground plane, while materials were FR4 for the substrate and vacuum for the space surrounding the SRR. Regarding excitations, terminal lumped ports were considered to simulate the 50 Ω SMA connectors soldered to the printed SRR. Parameter S_21_ versus frequency was obtained from 400 to 600 MHz to be corroborated with the measurement counterpart and see f_SR_ of S_21_ and compare it with f_MR_.

## 3. Results

### 3.1. SEM Images

**Figure 8:**
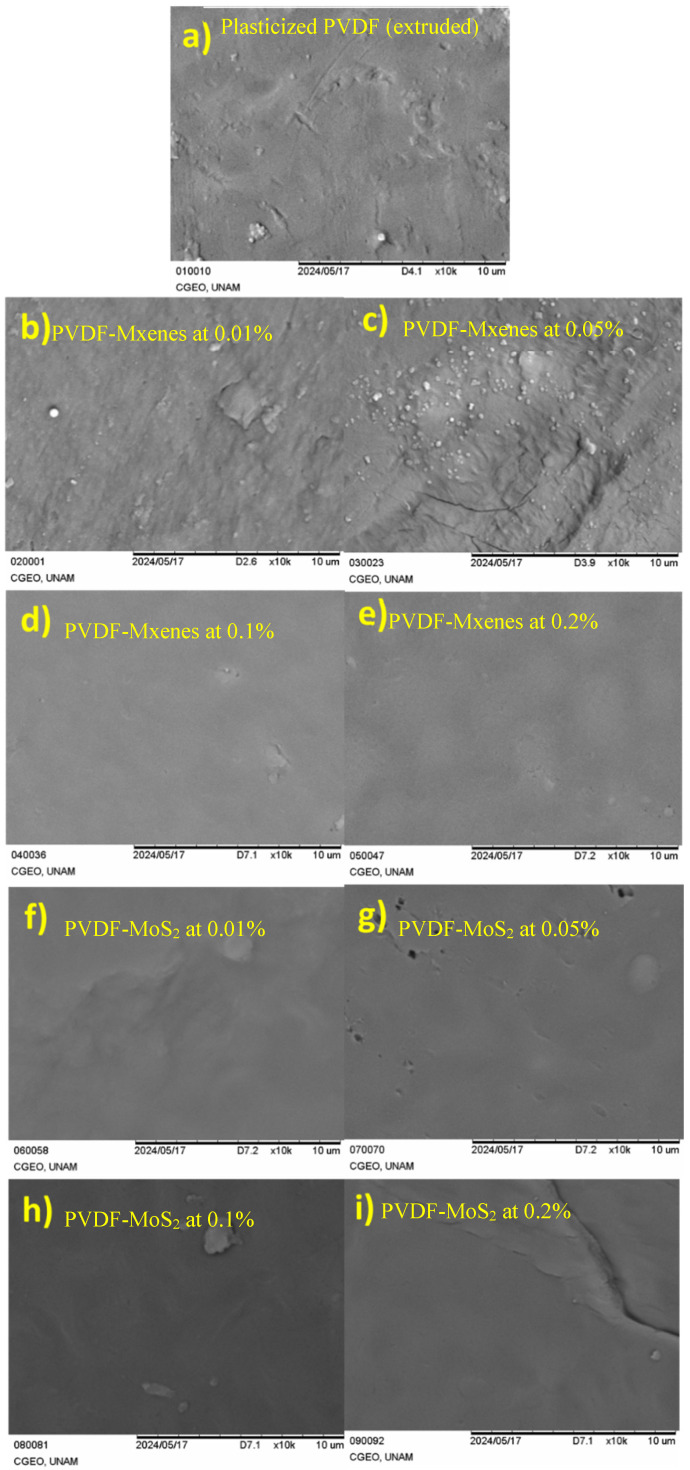
SEM micrographs for (**a**) plasticized PVDF (extruded), PVDF-MXenes at concentrations of (**b**) 0.01%, (**c**) 0.05%, (**d**) 0.1%, and (**e**) 0.2%, PVDF-MoS2 at concentrations of (**f**) 0.01%, (**g**) 0.05%, (**h**) 0.1%, and (**i**) 0.2%.

The analysis using SEM aims to identify changes in surface morphology, evaluate the homogeneity of nanomaterial dispersion, and compare structural differences among composites with varying nanomaterial concentrations. This study also seeks to correlate morphological observations with the electrical properties of the composites (samples located on the split of the SRR), providing valuable insights for their design and enhancement, with the results of these analyses shown in Figure 8. The SEM analysis revealed that the dispersion of nanomaterials is uniform in most of the composites, as they exhibit uniform surfaces. However, some material aggregations and small cracks were observed in certain composites, especially those with concentrations of 0.01% and 0.05% of MXenes. Conversely, composites with concentrations of 0.1% and 0.2% of MXenes showed very uniform surfaces. In contrast, composites with MoS_2_ displayed uniformity in surface properties for all concentrations. This analysis allowed us to evaluate the homogeneity of nanomaterial dispersion by determining how they are distributed within the polymer matrix and whether there are aggregates or areas with higher concentrations. The morphology analysis provides valuable insights into the successful production of the nanocomposites; however, it is not definitive in revealing the electrical properties studied later, aside from some noticeable deviations in certain tests, such as the 0.05% concentration of MXenes. Instead, the information gleaned from morphology analysis complements the results of the subsequent electrical characterizations (S_21_ around f_MR_ = 500.4 MHz) presented in the following sections. These electrical characterizations will offer a more comprehensive understanding of the relationship between the structure of the material and its electrical behavior, contributing to the overall assessment and refinement of these composites.

### 3.2. TEM Analysis of MXene and MoS_2_ Nanosheets

**Figure 9:**
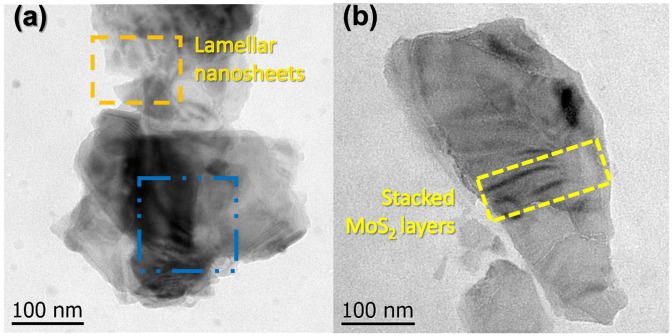
Transmission Electron Microscopy (TEM) images of the two-dimensional reinforcements: (**a**) MXenes, showing lamellar morphology and stacked nanosheets, and (**b**) exfoliated MoS_2_, where few-layer regions and stacked sheet structures can be observed.

Transmission electron microscopy (TEM) analysis reveals distinct morphological characteristics for the two-dimensional reinforcements, namely exfoliated MoS_2_ and MXene nanosheets (Figure 9). In the case of MoS_2_, the TEM images show plate-like structures with well-defined edges and localized regions of stacked layers (Figure 9a,b), which enable the identification of few-layer nanosheets with relatively uniform thickness. The presence of discernible stacking and partial edge exposure facilitates a qualitative assessment of the layered nature of exfoliated MoS_2_.

In contrast, MXene samples predominantly exhibit large-area, sheet-like morphologies with irregular edges and high overall transparency (Figure 9a,b). Pronounced contrast variations are observed across individual flakes, indicating local differences in the number of stacked layers, where highly transparent regions are associated with few-layer nanosheets and darker areas correspond to localized stacking. Owing to the extremely high lateral-to-thickness aspect ratio of MXene nanosheets, most flakes preferentially lie flat on the TEM grid, resulting primarily in plan-view images and limiting the availability of clear edge-view regions for direct thickness quantification.

Consequently, the nanometric thickness of MXene nanosheets is inferred qualitatively based on contrast variations, stacking features, and occasional folded or overlapping regions rather than from statistically averaged thickness measurements. Despite this limitation, the observed morphology characterized by high transparency, large lateral dimensions, and partial stacking is consistent with few-layer MXene nanosheets reported in the literature. Overall, the TEM observations confirm the two-dimensional, lamellar nature of both MoS**_2_** and MXenes, supporting their effective exfoliation and suitability as nanoscale reinforcements within the PVDF matrix [42,43,44].

### 3.3. S-Parameter Analysis

**Figure 10:**
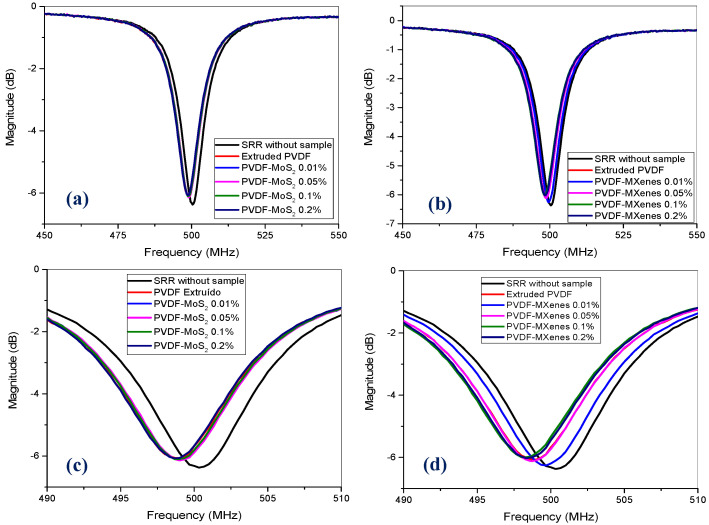
Parameter S_21_ for composite samples reinforced with (**a**) MoS_2_ and (**b**) MXenes. (**c**,**d**) Zooms from 490–510 MHz for both types of samples (**a**,**b**).

For the SRR electrical experiments, the scattering parameter S_21_ in dB was obtained as a sensing parameter for the sample concentration in a range of 400 to 600 MHz. Figure 10 presents the results of the characterization of the composites reinforced with both nanoparticles: MoS_2_ and MXenes. The close-ups in Figure 10c,d show f_MR_ of S_21_ in the presence of samples of the composites of both types of materials, which occurred at around 500.4 MHz. In addition to the comparison between samples with different nanoparticle concentrations, the scattering diagrams (S_21_) include the resonant ring without a sample. From this comparison (Figure 10c,d), the shift in the graphs for the composite samples is noticeable. In the case of materials with MoS_2_, the shift is similar for all samples, while it is more dispersed among materials reinforced with MXenes. In this work, four types of concentrations were considered, so having a wider range of concentrations would be a good experiment to observe a more generalized behavior of the sensor in the near future.

**Figure 11:**
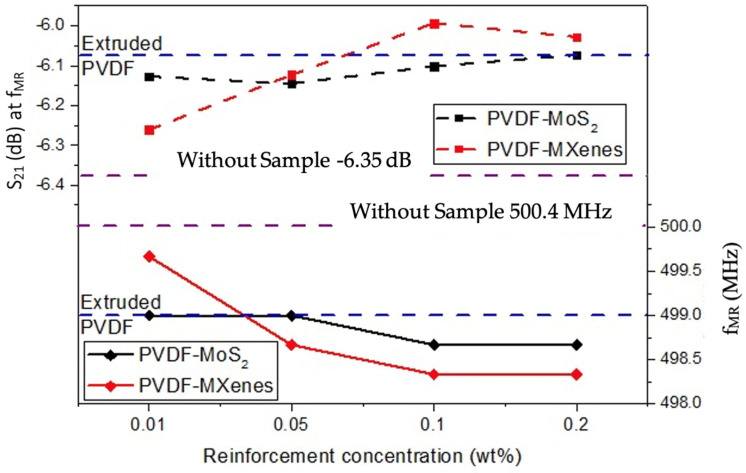
“S_21_ (dB)” and “f_MR_” for the SRR containing PVDF-based reinforced composites.

Figure 11 contains the results representing the level in dB of parameter S_21_ as a function of the concentration level for each material placed as a sample in the SRR split. In addition, the frequency f_MR_ corresponding to each concentration level of the composites is observed. It is observed that there is no linear behavior, neither for S_21_ (dB) nor for f_MR_ when varying concentrations; however, a trend is identified in which S_21_ (dB) increases as the concentration of nanoparticles increases. On the other hand, an opposite effect is observed in f_MR_, which decreases as the concentration of nanoparticles increases. As a reference, the blue and purple dotted lines represent the values of S_21_ (dB) at f_MR_ for PVDF extruded (0%) as a sample and for SRR without a sample, respectively.

**Figure 12:**
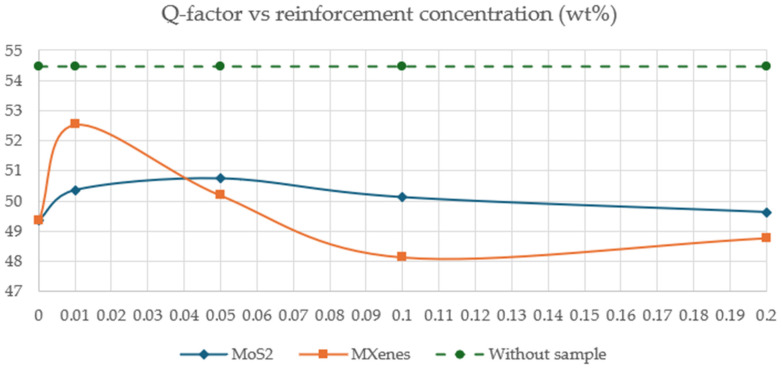
Q-factor in terms of reinforcement concentration.

Information in Figure 9 regarding S_21_ leads to the Q-factor for the SRR without and with the samples that were considered. For the SRR without a sample, Q = 54.48, while for the sample corresponding to extruded PVDF (0% either of MoS_2_ or MXenes), Q = 49.35. For samples with concentrations of MoS_2_ at 0.01, 0.05, 0.1, and 0.2%, values of Q were as follows: 50.37, 50.76, 50.13, and 49.63, respectively, while concentrations of MXenes at the same percentages were as follows: 52.54, 50.19, 48.14, and 48.78, respectively. Figure 12 shows the plot of the Q-factor in terms of reinforcement concentration of both MoS_2_ (diamond-shaped markers) or MXenes (square-shaped markers). The plot with circular markers corresponds to SRR without a sample.

### 3.4. Simulated S_21_ (dB) vs. Measured S_21_ (dB)

**Figure 13:**
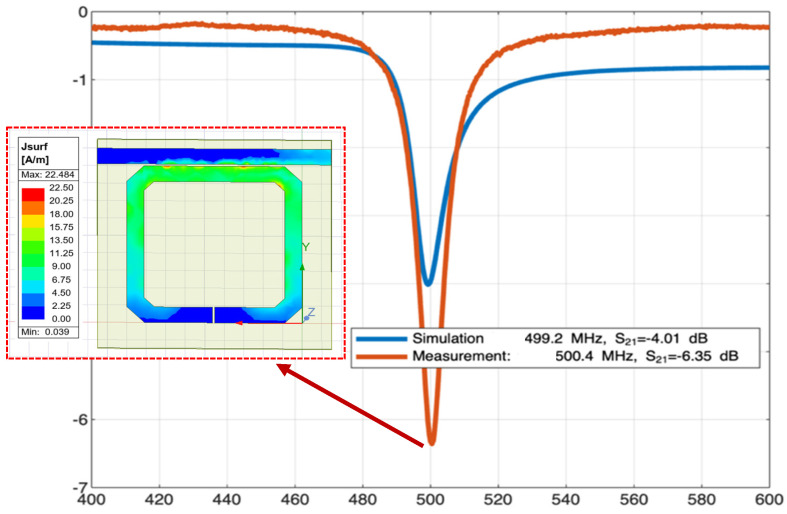
S_21_ (dB) for SRR without sample: simulated vs. measured where f_MR_ and f_SR_ are shown.

Figure 13 shows parameter S_21_ in dB for the SRR without a sample (simulated vs. measured); likewise, the simulated surface current density at 499.2 MHz is plotted. It can be noticed in the simulation that f_SR_ is at 499.2 MHz with S_21_ = −4.01 dB, while in measurement, S_21_ was −6.35 dB at f_MR_ = 500.4 MHz, making the simulation results consistent with the measurement results. It is important to mention that the lumped-element equivalent resonances f_RL_ = f_RL_′ of the SRR are 505.96 MHz as mentioned previously.

In terms of the correlation with the S_21_ (dB) parameter, it is observed that, in general, the value of S_21_ (dB) increases as the reinforcement concentration grows, both for MoS_2_ and MXenes. However, the change is more pronounced in the case of PVDF-MXenes, suggesting that MXenes have a more significant effect on the electromagnetic properties of the material. Specifically, for PVDF-MoS_2_, S_21_ (dB) varies from −6.12688 dB (at 0.01%) to −6.07278 dB (at 0.2%), while for PVDF-MXenes, S_21_ (dB) goes from −6.26115 dB to −6.02814 dB. While these variations are small, the trend suggests that the inclusion of MXenes has a greater impact on improving the electromagnetic properties, due to the higher influence of the nanoparticles on the structure of the material.

A key finding of this study is that the addition of MXenes has a stronger effect on the electromagnetic and structural properties of PVDF compared to MoS_2_. This is reflected in changes to the measured resonance frequency f_MR_ and S_21_ (dB) parameters, indicating that the presence of MXenes significantly modifies the electromagnetic behavior of the material as expected and due to the stronger interaction between the nanoparticles and the polymer matrix. As the concentration of MXenes increases, a more pronounced variation in the resonance frequency (f_MR_) and the S_21_ (dB) value is observed, indicating a notable influence of these nanoparticles on the electromagnetic properties of the material.

This behavior is attributed to the inherent characteristics of MXenes, such as their high conductivity and two-dimensional structure, which facilitate stronger interactions with the electromagnetic field. In contrast, for MoS_2_, which is a semiconductor with a much lower conductivity, leading to a weaker but still measurable interaction, although also showing variations of S_21_ (dB) and f_MR_, suggesting that the electromagnetic properties of MoS_2_ do not significantly modify the electromagnetic behavior of the composite compared to MXenes. This demonstrates the sensor’s capability not only to detect the presence of nanoparticles but also to discriminate between materials with distinct electromagnetic signatures.

Finally, it is worth mentioning that after a meticulous search, Table 3 containing the main findings reported in the scientific literature regarding the topic of this article is presented. The articles regarding detection of concentrations of nanoparticles included Table 3, which reported a microwave sensor (device), the analyte, the concentration range, and the sensitivity whether in dB/ppm or in Hz/ppm depending on S-parameter level or frequency shift, respectively. In Table 3, our study is included in the last row considering linear regression for slope in Hz/ppm. Our sensitivities are like, and even greater than, those considered in Table 3. The comparison table shows results of other relevant studies related and respected, although they use other types of sensors regarding shape, size and sensing method.

## 4. Discussion

In general, although variations in concentrations of MoS_2_ and MXenes were very similar (0.01% to 0.2%), it was observed that, both for MoS_2_ and MXenes, as concentration increases, S_21_ (dB) also increases, while f_MR_ decreases. The dynamic range for S_21_ (dB) variation was about −0.3 dB wide, while the corresponding range for f_MR_ variations was about 1.25 MHz (Figure 10). In simple words, the resonance frequency f_MR_ of S_21_ (dB) starts at (500.4 MHz, −6.35 dB) and goes to the left and up as concentrations increase. From Figure 10c,d, it is clear that MXenes concentrations cause more noticeable shifts in S_21_ (dB) plots.

The presence of MXenes has a more noticeable effect on the electromagnetic properties compared to MoS_2_, suggesting that MXenes, due to their unique conductivity and 2D structure, enhance the response of the material to electromagnetic waves. These findings highlight the potential for tuning the properties of the material by adjusting nanoparticle concentrations, which could be valuable for further optimization in applications involving electromagnetic wave interactions, such as electromagnetic shielding or RF/microwave sensors.

## 5. Future Work

While the results successfully demonstrate the sensor viability as proof-of-concept, it is acknowledged that this study did not include a systematic analysis of measurement repeatability or a determination of the detection limit. Future work should focus on these crucial performance metrics, including repetitive measurements to calculate standard deviation and a comprehensive study to establish the sensor limit of detection, which will be essential for any practical application. Likewise, future work is contemplated to estimate the complex permittivity of materials under test (samples of PVDF-based nanocomposites).

## Figures and Tables

**Table 1:** SRR dimensions.

Description	Dimensions (mm)
Substrate dimensions (l_PCB_ × l_PCB_)	60 × 60 × 1.5
MTL dimensions (w × l_PCB_)	4.5 × 60
Trace width forming the ring (w)	4.5
External SRR dimensions	45 × 45
Split dimensions (g × w)	0.5 × 4.5
Distance between the SRR and the MTL (S)	0.5

**Table 2:** Resonance frequencies and LC lumped elements for LEC and LESC models.

Circuit	Inductance (nH)	Capacitance (pF)	Resonance in MHz at:	Equation
LEC	Ls = 68.57	C_s_ = 1.443	505.96	$f_{RL}=\frac{1}{2\pi \sqrt{L_{S}C_{S}}}$
LESC	L_s_′ = 188.98	C_s_′ = 0.525	505.96	${f_{RL}}^{\prime }=\frac{1}{2\pi \sqrt{L_{S}^{\prime }C_{S}^{\prime }}}$

Note that f_RL_ and f_RL_′ are the same (red plot in Figure 7) and valid for LEC and LESC.

**Table 3:** Comparison of some of the most relevant findings regarding. microwave sensor for nanoparticle detection.

Sensor Material	Structure	Analyte	Concentration Range	Sensitivity
Ti_3_C_2_Tₓ MXene	SRR [45]	NO_2_	2–10,000 ppb	98.66 mdB/ppm
Ti_3_C_2_Tₓ MXene	Planar sensor [46]	Acetone	1–500 ppm	17.85 kHz/ppm
MoS_2_/MoOₓ Nanoflakes	Antenna [47]	VOCs (Methanol)	1000–8000 ppm	892 Hz/ppm
PVDF-Mxene and PVDF/MoS_2_	SRR [our work]	Mxene-MoS_2_	0–2000 ppm	520.2399 Hz/ppm–192.9535 Hz/ppm

## Data Availability

The data is available upon request.

## Abbreviations

The following abbreviations are used in this manuscript:

PVDF	Polyvinylidene fluoride
UHF	Ultra-high Frequencies
MXenes	MXenes
MoS_2_	Molybdenum disulfide
NPs	Nanoparticles
EMI	Electromagnetic Interference
AFM	Atomic Force Microscopy
TEM	Transmission Electron Microscopy
SRR	Split-Ring Resonator
SEM	Scanning Electron Microscopy
VNA	Vector Network Analyzer
HFA	Hydrofluoric Acid
DMSO	Dimethyl Sulfoxide
IPA	Isopropyl Alcohol
PCB	Printed Circuit Board
MTL	Microstrip Transmission Line
SMA	SubMiniature version A
MUT	Material Under Test
SUT	Samples Under Test
LoRa	Long Range
SOLT	Short-Open-Load-Through
IoT	Internet of Things
LEC	Lumped-Element Circuit
LESC	Lumped-Element Simplified Circuit
QUCS	Quite Universal Circuit Simulator
AEDT	Ansys Electronics Desktop^®^
